# Systemic lupus erythematosus–driven accelerated atherosclerosis: the immune–metabolic–vascular axis and therapeutic implications

**DOI:** 10.3389/fimmu.2026.1841513

**Published:** 2026-06-10

**Authors:** Meiwei Jiang, FengQi Zhang, MinZhe Ren, ZhiYu Li, ZhiJun Xie, Jing Sun

**Affiliations:** 1 The Second School of Clinical Medicine, Zhejiang Chinese Medical University, Hangzhou, China; 2 Innovative Institute of Chinese Medicine and Pharmacy, Shandong University of Traditional Chinese Medicine, Jinan, China; 3 The First Affiliated Hospital of Zhejiang Chinese Medical University (Zhejiang Provincial Hospital of Chinese Medicine), Hangzhou, China; 4 College of Basic Medical Science, Zhejiang Chinese Medical University, Hangzhou, China; 5 Jinhua Research Institute, Zhejiang Chinese Medical University, Jinhua, China; 6 The Affiliated Lishui Hospital of Traditional Chinese Medicine, Zhejiang Chinese Medical University, Lishui, China

**Keywords:** atherosclerosis, dysfunctional HDL, immunometabolism, neutrophil extracellular traps, systemic lupus erythematosus, type I interferon

## Abstract

Patients with systemic lupus erythematosus (SLE) are at markedly increased risk of premature atherosclerosis (AS) and atherosclerotic cardiovascular disease (ASCVD), and this excess risk is not fully explained by traditional Framingham factors. Increasing evidence suggests that SLE does not merely coexist with AS; rather, persistent immune activation and immunometabolic dysregulation reshape the vascular microenvironment toward endothelial dysfunction, lipoprotein impairment, maladaptive myeloid activation, and immunothrombosis. This review synthesizes current epidemiologic, mechanistic, and translational evidence supporting an immune–metabolic–vascular framework for SLE-accelerated AS. We focus on four interconnected processes: (1) type I interferon (IFN-I)-associated endothelial injury and defective vascular repair; (2) neutrophil extracellular traps (NETs) and oxidative modification of high-density lipoprotein, contributing to dysfunctional or pro-inflammatory HDL; (3) monocyte/macrophage immunometabolic reprogramming, which favors foam-cell formation and inflammasome activation; and (4) T- and B-cell metabolic disequilibrium, which sustains vascular inflammation and autoantibody-driven immune injury. Across these pathways, metabolic rewiring appears to function not merely as a parallel phenomenon, but as a shared amplifier linking systemic autoimmunity to lesion-level vascular progression. Recognizing these shared checkpoints has therapeutic implications. These observations suggest that future strategies may need to integrate upstream metabolic resetting, midstream immune-specific blockade, and downstream lipid or vascular-wall protection, rather than relying solely on lipid lowering or broad immunosuppression. However, most available evidence remains confined to mechanistic studies, biomarker readouts, or surrogate vascular endpoints, and dedicated trials with plaque or cardiovascular event outcomes are still needed.

## 1 Introduction

Atherosclerotic cardiovascular disease (ASCVD) is classically regarded as a progressive disorder associated with aging, metabolic decline, and the loss of sex-related vascular protection, with risk rising substantially after menopause in women and across midlife in men (1). In systemic lupus erythematosus (SLE), however, this conventional epidemiologic pattern is substantially altered: young women of childbearing age, who would otherwise be relatively protected from premature cardiovascular disease, become a high-risk population for early vascular events (2). A landmark cohort study by Manzi et al. showed that women with SLE aged 35–44 years had a myocardial infarction risk more than 50-fold higher than that of age-matched women from the Framingham Offspring Study (3), and subsequent work demonstrated that traditional Framingham factors do not fully account for this excess cardiovascular burden (4).

This observation is notable because premenopausal estrogen is generally considered vasculoprotective in the general population, yet in SLE such protection appears attenuated or overridden by disease-specific inflammatory and immune mechanisms; at the same time, estrogens may also play a permissive role in lupus susceptibility, underscoring the complex sex-specific biology of vascular risk in SLE (5–7). Clinical and imaging studies further indicate that subclinical endothelial dysfunction and atherosclerotic plaque burden can be detected in some patients even when conventional lipid measures appear unremarkable, suggesting that vascular injury in SLE cannot be understood solely through a lipid-centric lens. Although chronic systemic inflammation has long been invoked to explain accelerated atherosclerosis in SLE, this explanation is incomplete, particularly because standard anti-inflammatory therapies such as glucocorticoids may themselves worsen metabolic risk (8, 9).

Increasingly, attention has therefore shifted upstream toward immunometabolism. Emerging transcriptomic and immunometabolic studies suggest that circulating monocytes, macrophages, and lymphocyte subsets in SLE acquire pro-inflammatory and maladaptive metabolic programs before or during tissue entry, potentially predisposing the vascular wall to oxidative stress, defective repair, and lipid retention (10). In this context, SLE may be conceptualized not merely as a coexisting comorbidity or additive risk factor, but as a disease-specific contributor to premature vascular injury (11). This review therefore examines how immunometabolic reprogramming links systemic autoimmunity to local atherogenesis and how this framework may refine future prevention and treatment strategies.

This is a narrative review rather than a systematic review. We searched PubMed, Web of Science, and Scopus from database inception to March 2026 using combinations of the following terms: “systemic lupus erythematosus,” “atherosclerosis,” “cardiovascular disease,” “immunometabolism,” “type I interferon,” “neutrophil extracellular traps,” “dysfunctional HDL,” “monocyte metabolism,” “T cell metabolism,” and “B cell metabolism.” We included peer-reviewed original studies, clinical trials, translational studies, animal experiments, and high-quality reviews that addressed immune, metabolic, vascular, or therapeutic mechanisms related to SLE-associated atherosclerosis. Non-English articles, conference abstracts without full text, studies unrelated to SLE or atherosclerosis, and reports without mechanistic or clinical relevance were excluded. Seminal epidemiological studies were also included to provide historical context.

## 2 Immunity and metabolism in systemic lupus erythematosus

### 2.1 Overview of immune inflammation and metabolic disorders in SLE

The pathogenesis of SLE is traditionally summarized as a loss of autoantigen tolerance. This leads to the production of pathogenic autoantibodies and organ damage mediated by immune complex deposition (12). The production of autoantibodies is indeed a hallmark feature and core effector stage of SLE. However, B cells do not drive the disease alone. An immune network jointly determines the amplification of inflammation and the persistence of tissue damage (13) (14). This network consists of T cells, neutrophils, plasmacytoid dendritic cells (pDCs), and monocytes and macrophages.

Accumulating preclinical and clinical evidence has established that immune system dysfunction in SLE is intrinsically linked to profound alterations in intracellular metabolic states (15). Specifically, the abnormal activation state of immune cells relies heavily on a systematic remodeling. This remodeling occurs in their energy metabolism and biosynthetic pathways. At the same time, a disordered metabolic microenvironment forms through mitochondrial dysfunction, reactive oxygen species (ROS) accumulation, and key metabolic intermediates. This environment further promotes the continuous activation of the type I interferon (type I IFN) pathway. It also drives the abnormal differentiation and response of autoreactive B cells and T cells (16) (17). Therefore, abnormal immune activation and metabolic dysregulation should not be viewed as fully independent processes. Rather, they represent interconnected components of a shared pathophysiological network with reciprocal regulatory interactions (18). This perspective extends the pathogenesis of SLE beyond a purely immunological framework and highlights immunometabolism as an organizing principle for understanding disease persistence and tissue injury.

In SLE, pDCs produce large amounts of type I IFN through nucleic acid recognition receptors. These are especially endosomal Toll like receptors 7 and 9 (TLR7 and TLR9). This process is a major source of the interferon signature (19). At the human genetic level, researchers have proven that TLR7 gain of function variants can monogenically trigger a lupus like autoimmune phenotype. This directly supports a specific pathogenic pathway in human disease. This pathway moves from abnormal nucleic acid recognition to enhanced type I IFN signaling and finally to autoimmune initiation (20). Within this inflammatory microenvironment, metabolic sensors are activated. These include the mechanistic target of rapamycin (mTOR) and hypoxia inducible factor 1 alpha (HIF-1α). This activation promotes a high-demand metabolic state in immune cells (21). It provides a common upstream starting point for several typical pathogenic pathways. These pathways include the T cell mTOR axis, the mitochondrial nucleic acid sensing axis, and the lipoprotein functional deterioration axis.

### 2.2 Typical pathways

#### 2.2.1 T cell-mTOR pathway

Increasing evidence shows a specific state in CD4^+^ T cells among SLE patients. These cells exhibit a state of metabolic overactivation (22). Importantly, this state is not a simple decrease in oxidative phosphorylation (OXPHOS). Instead, it often presents as a simultaneous increase in both glycolysis and mitochondrial oxidative metabolism. This occurs alongside mitochondrial hyperpolarization (MHP) and a higher ROS burden (23). These features suggest a specific functional state for mitochondria. These mitochondria appear to operate in a high-output but stress-prone and functionally unstable state (24). During this process, mTOR acts as a critical hub (25). This is especially true for mTORC1. It functions as a nutrient and energy sensor to integrate signals from amino acids, glucose, and oxygen. It drives the T cell metabolic program toward an effector mode. This mode relies heavily on glycolysis and biosynthesis. Furthermore, it promotes T helper 17 (Th17) cell differentiation and inhibits regulatory T cell (Treg) differentiation. It achieves this through metabolic checkpoints related to HIF-1α. Consequently, this exacerbates peripheral tolerance breakdown (24).

Translational evidence further suggests that this pathway may be therapeutically modifiable. This highlights its potential as a therapeutic target. A randomized double blind trial involving human subjects tested N-acetylcysteine (NAC). This trial suggested that NAC may improve SLE disease activity, at least partly through inhibition of T-cell mTOR activation (26). Furthermore, an open label phase I/II study evaluated sirolimus or rapamycin. This study also showed a decrease in disease activity. It additionally indicated a correction in T cell lineage imbalances (27). Murine models provide further mechanistic insights through dual metabolic targeting. For instance, Yin et al. observed concurrent upregulation of glycolysis and mitochondrial metabolism in CD4^+^ T cells of lupus-prone mice, demonstrating that combining metformin with 2-deoxy-D-glucose (2DG) effectively corrected this aberrant metabolic program. Metformin inhibits mitochondrial complex I and reduces mitochondrial metabolic drive and ROS. 2DG inhibits glycolysis. This combined intervention attenuated the abnormal metabolic program and improved lupus-like disease phenotypes in experimental models (28) (29). On a clinical level, randomized controlled trials tested metformin in non-diabetic SLE populations. These trials provided a feasibility signal for “drug repurposing” (30). However, researchers still need to validate these findings. They require larger sample sizes and trials focusing on vascular or event driven endpoints. Overall, mTOR and its downstream immunometabolic programs form a critical pathway. This pathway connects nutrient sensing to autoimmune activation (31). It lays the foundation for discussing metabolic targeted therapies later. Examples include the regulation of the mTOR and AMP-activated protein kinase (AMPK) axis. It also helps explain the upstream driving mechanisms behind the accelerated AS risk associated with SLE.

#### 2.2.2 Mitochondria-cGAS-STING pathway and type I interferon axis

Mitochondrial dysfunction represents an important entry point for understanding immunometabolic dysregulation in SLE. Immune cells in SLE often show abnormal mitochondrial membrane potentials. This is especially true for T cells and neutrophils. These cells also display increased reactive oxygen species (ROS) and enhanced mitochondrial stress responses (32). These changes affect cellular energy metabolism homeostasis. They may also increase the likelihood of mitochondrial DNA (mtDNA) release into the cytoplasm or extracellular space. This process provides a continuous stimulus for nucleic acid sensing pathways in the innate immune system (16). The cycle of mitochondrial stress, nucleic acid release, and interferon amplification may be best interpreted as an important disease-maintaining mechanism in SLE. It is not necessarily a single pathogenic starting point common to all patients. Double-stranded DNA (dsDNA) can appear in the cytoplasmic environment. When this happens, cyclic GMP-AMP synthase (cGAS) recognizes the dsDNA and produces 2’3’-cGAMP. This molecule then activates the stimulator of interferon genes (STING). STING induces the expression of IFN-α/β and interferon-stimulated genes (ISGs) through the TBK1-IRF3 axis. This cascade helps form and maintain the interferon signature (33). The recognition of cytosolic dsDNA by cGAS relies on the backbone structure of the DNA molecule. It does not depend on specific CpG dinucleotide sequences. Mechanistic studies have systematically verified this fact (33). In contrast, TLR9 is an endosomal nucleic acid sensing receptor. It specifically recognizes unmethylated CpG DNA (34). TLR9 performs a different immune triggering function within the classical pattern recognition receptor system.

Neutrophil extracellular traps (NETs) provide a highly interferogenic source of nucleic acids in SLE. A 2016 study by Lood et al. in Nature Medicine demonstrated a key finding (35). NETs are rich in oxidized mtDNA. This oxidized mtDNA has a stronger ability to induce interferons and correlates with lupus-like phenotypes. This suggests that mitochondrial stress is not just an intracellular metabolic event. It may also help maintain the chronic inflammatory environment driven by type I IFN. This occurs through nucleic acid release and innate immune sensing mechanisms.

The role of the cGAS-STING pathway is not simply uniform across different models and stages of lupus pathology (36). Multiple studies show that inhibiting STING reduces type I IFN related responses and autoimmune lesions. Researchers achieved this through gene knockout or pharmacological intervention. This indicates that this axis contributes to the disease in specific contexts (37). However, other reports present a different view. The cGAS-STING pathway is not an essential driver of systemic autoimmunity in certain SLE models. It even shows complexity or inverse effects in some of these models (38). This model- and stage-dependent variability suggests that cGAS-STING signaling may contribute to selected SLE endotypes rather than to all disease contexts (39). The cGAS-STING pathway likely acts as a key node for amplifying nucleic acid driven signals in specific endotypes. We should not generalize its role to all patients with SLE. Therefore, future strategies targeting this pathway should rely on biomarker stratification. This includes inflammatory markers like circulating mtDNA and the interferon signature. It also involves assessing mitochondrial stress levels and NET burden. This approach will help evaluate treatment efficacy and safety in more precise patient subgroups.

#### 2.2.3 Lipid metabolism and atherosclerosis pathway

With improved control of active nephritis and infections, premature atherosclerosis and cardiovascular events have become major contributors to long-term morbidity and mortality in patients with SLE. This accelerated vascular phenotype presents a clinically important discrepancy between conventional risk prediction and observed vascular burden. Patients often lack traditional Framingham risk factors like significant obesity or stubborn hypertension. However, they still experience rapid plaque progression (40). This observation suggests that qualitative alterations in lipid metabolism may contribute to SLE-associated vascular disease. A qualitative change in lipid metabolism drives SLE vascular disease, rather than a simple quantitative change. Thus, lipoprotein functional heterogeneity may represent an important contributor to SLE-associated vascular injury. The absolute levels of high-density lipoprotein cholesterol (HDL-C) may appear normal in some patients. Even then, their HDL particles often undergo pathological remodeling (41). HDL can switch from an anti-inflammatory and antioxidant state to a pro-inflammatory state. This happens under inflammatory and oxidative stress conditions. Basic research clearly describes this phenomenon as acute-phase remodeling (41). McMahon et al. further proposed and validated this concept in SLE populations. They showed that pro-inflammatory HDL (piHDL) correlates significantly with subclinical AS (42).

Paraoxonase-1 (PON1) is an important part of the antioxidant function related to HDL. A decrease in PON1 activity correlates with AS risk in patients with SLE. This provides a quantifiable enzymatic clue for impaired HDL function (43) (44). Oxidized lipids and impaired cholesterol handling play a role from the perspective of plaque mechanisms. They promote macrophage foam cell formation and amplify sterile inflammation. In particular, cholesterol crystals can activate the NLR family pyrin domain containing 3 (NLRP3) inflammasome. This activation participates in the development and progression of AS (45). This provides mechanistic support for a pathway linking oxidized lipid burden to plaque inflammation. It shows how an increased oxidized lipid burden amplifies plaque inflammation. On a clinical translation level, simply lowering low-density lipoprotein (LDL) is not enough. It cannot completely offset the residual cardiovascular risk in SLE. The Lupus Atherosclerosis Prevention Study (LAPS) illustrates this translational limitation. Two years of atorvastatin treatment did not significantly improve subclinical indicators in patients with SLE. These indicators include coronary calcium, intima-media thickness (IMT), and plaque (46). The Atherosclerosis Prevention in Pediatric Lupus Erythematosus (APPLE) trial also failed to meet its primary endpoint. This endpoint was carotid intima-media thickness (CIMT) in pediatric SLE (47). These results align well with the risk model driven by the immune-metabolic-vascular axis. This means that addressing SLE-related AS requires simultaneous intervention. Effective risk modification may require coordinated targeting of inflammation, immunometabolism, and lipid-related pathways. Relying solely on traditional lipid lowering therapies is insufficient.

### 2.3 The pathological profile of SLE

In summary, the pathological profile of SLE goes beyond simple immune dysregulation. It represents a pathogenic positive feedback loop between immune inflammation and metabolic reprogramming. This metabolic deviation is not merely a passive byproduct of immune activation; rather, it provides the essential bioenergetic substrates and intracellular signaling prerequisites to sustain chronic systemic inflammation (48). Shared metabolic nodes include mTOR activation (49), mitochondrial stress, and lipid peroxidation. Systemic autoimmunity uses these shared nodes to break through anatomical barriers. It then settles in target tissues like the vascular wall and causes persistent damage (50). These observations support the conceptual importance of the immune–metabolic–vascular axis in SLE-associated vascular disease. Doing so marks a fundamental shift in our understanding of SLE comorbidities. This perspective shifts the interpretation of SLE comorbidities from passive associations toward mechanistically linked disease processes. This framework may also help identify metabolic targets for future strategies aimed at attenuating accelerated AS.

## 3 Immune response in atherosclerosis

The immune response in AS is a chronic inflammatory process. Both innate and adaptive immunity participate in this ongoing condition. Current mainstream views suggest a new understanding of the key initiating event in AS. It is not simply lipid accumulation. Instead, the process begins with the retention of apolipoprotein B (ApoB) rich lipoproteins in the arterial intima. These lipoproteins then undergo subsequent modifications. These changes trigger endothelial activation, immune cell recruitment, and local sterile inflammation. Ultimately, they determine the risk of plaque progression and rupture. The conceptual framework of AS has shifted from a lipid-storage model toward a chronic inflammatory vascular disease model. We no longer view it merely as a lipid storage disease. We now recognize it as a chronic inflammatory vascular disease. Abnormal lipids provide the substrates and trigger signals. Meanwhile, various immune cells shape the plaque composition at different stages. These cells include monocytes/macrophages, dendritic cells (DCs), neutrophils, and T/B cells. They also determine the intensity of inflammation, the stability of the fibrous cap, and the risk of thrombotic complications (51).

### 3.1 Triggers and early events

High cholesterol and oxidized low-density lipoprotein (oxLDL) combine with changes in blood flow shear stress. These factors activate endothelial cells. The activated cells then express adhesion molecules. This process recruits monocytes and lymphocytes into the vascular wall. Furthermore, the immune system can recognize the modified lipids themselves as danger signals. Endothelial and local cells sense this danger through pattern recognition receptors (PRRs). These receptors include Toll-like receptors (TLRs) and NLRP3. Their activation triggers inflammatory cascades, such as the interleukin-1 beta (IL-1β) and interleukin-18 (IL-18) pathways. These early innate signals are important for lesion initiation and inflammatory propagation. They initiate the vascular lesions and sustain the ongoing inflammation (52, 53).

Innate immunity plays a core role across all stages of AS. This includes its initiation, progression, and the final unstable phase (54). This system relies on PRRs to rapidly recognize modified lipids and damage-associated molecular patterns (DAMPs). It can initiate inflammatory responses independently of antigen-specific immunological memory. It then uses cytokines, chemokines, and effector molecules. These factors drive the continuous recruitment of immune cells and foam cell formation. They also cause the necrotic core to expand and the fibrous cap to thin.

### 3.2 Innate immune response

The innate immune response is a core driving mechanism for the occurrence and progression of AS (55). It senses modified lipids and DAMPs through PRRs. This recognition quickly starts the inflammatory cascade. Importantly, cholesterol crystals can activate the NLRP3 inflammasome. This activation promotes the maturation and release of IL-1β and IL-18. Researchers consider this mechanism a key molecular hub. It connects lipid metabolism disorders to the amplification of inflammation (56). On a pathological level, innate immune cells experience continuous activation. This is especially true for macrophages and DCs. This activation drives early fatty streak formation and foam cell accumulation. In later stages, these cells secrete matrix metalloproteinases and inflammatory cytokines. They also promote cell apoptosis. These actions accelerate the thinning of the fibrous cap and increase plaque instability. Therefore, innate immune imbalance persists throughout all stages of AS. This includes its initiation, progression, and acute event phases.

#### 3.2.1 Macrophages

In classical atherosclerosis, circulating monocytes infiltrate the intima and gradually differentiate into lipid-laden foam cells (57). Recent single-cell transcriptomics (scRNA-seq) have revealed that these plaque macrophages exist as a continuous spectrum, encompassing resident-like, pro-inflammatory, and TREM2-high foam-like subsets (58). In contrast to the gradual lipid accumulation typical of conventional AS, monocytes and macrophages in SLE may be metabolically and inflammatory primed in the periphery before vascular entry. As discussed earlier, systemic type I IFN and heightened glycolysis prime these monocytes, thereby increasing their responsiveness to oxidized lipids after vascular entry and potentially promoting inflammasome-driven foam-cell formation.

#### 3.2.2 Dendritic cells

DCs act as professional antigen-presenting cells that orchestrate T cell polarization within the plaque microenvironment (54, 59). However, in the context of SLE, the dendritic cell compartment—particularly plasmacytoid DCs (pDCs)—may exert an upstream regulatory role. Through persistent nucleic acid sensing and sustained type I IFN production, they not only disrupt systemic tolerance but also directly fuel the endothelial metabolic stress and monocyte pre-programming that precipitate accelerated atherogenesis.

#### 3.2.3 Neutrophils

Increasing evidence supports the involvement of neutrophils in early atherogenesis and later thrombotic complications (60). They release proteases like myeloperoxidase (MPO) that aggravate endothelial damage (61). More importantly, neutrophil extracellular traps (NETs) provide a physical scaffold for platelet adhesion, promoting immunothrombosis (62). While NETs act as inflammatory amplifiers in general AS, they play a more disease-specific amplifying role in the SLE microenvironment. In SLE, impaired NET clearance may create a substantial oxidative burden that contributes to the conversion of protective HDL into pro-atherogenic piHDL, thereby functioning as a disease-specific amplifier of vascular injury.

### 3.3 Adaptive immune response

Adaptive immunity features antigen specificity and immunological memory. Adaptive immunity exerts dual, opposing roles in atherogenesis (54). It simultaneously promotes inflammation and exerts inhibitory effects. Overall, the T helper 1 (Th1) response serves as a clear pro-atherosclerotic immune axis. Meanwhile, the Treg response correlates with inflammation suppression and plaque stability. The roles of Th17 and CD8+ T cells are more complex. Their functions depend heavily on the specific disease stage and the local microenvironment (63).

#### 3.3.1 T lymphocytes

CD4^+^ T helper cells represent the most thoroughly studied subpopulation in plaque adaptive immunity. In general atherosclerosis, Th1 cells drive plaque progression via IFN-γ secretion, which enhances the inflammatory programs of macrophages and impairs collagen repair responses in smooth muscle cells (SMCs), potentially weakening fibrous cap stability (64). In contrast, regulatory T cells (Tregs) usually suppress the inflammatory activity of effector T cells and myeloid cells through pathways involving interleukin-10 (IL-10) and transforming growth factor-beta (TGF-β), thereby maintaining immune tolerance and plaque stability (65). Additionally, while the exact role of Th17 cells remains complex and context-dependent (66), cytotoxic CD8^+^ T cells also appear in plaques and likely contribute to sustained vascular damage through direct cytotoxicity and cytokine pathways, though further human translational evidence is still needed (67). In SLE, the T-cell compartment is shaped by systemic mTORC1 hyperactivation and associated metabolic remodeling. These metabolically remodeled T cells are biased toward pro-inflammatory Th1 and Th17 phenotypes and exhibit a profound defect in protective Treg generation. This metabolic rewiring may markedly disturb the typical immune balance described above, thereby limiting regulatory constraints on local vascular inflammation once these aberrant T cells colonize the arterial wall.

#### 3.3.2 B lymphocytes

In general atherogenesis, B-1 cells exert a protective effect by secreting natural IgM antibodies to neutralize oxidized lipids, whereas conventional B-2 cells generally show a pro-atherosclerotic tendency (68). In SLE-driven AS, however, this protective balance is substantially disrupted, and the humoral axis becomes aberrantly hyperactive. Metabolically hyperactive B cells continuously generate pathogenic autoantibodies (such as antiphospholipid antibodies),potentially transforming atherosclerotic lesions into sites of autoantigen-associated chronic immune activation that persistently amplifies local vascular inflammation and thrombotic risk.

## 4 Systemic lupus erythematosus-driven accelerated atherosclerosis: mechanistic and translational evidence for the immune–metabolic–vascular axis

**Figure 1:**
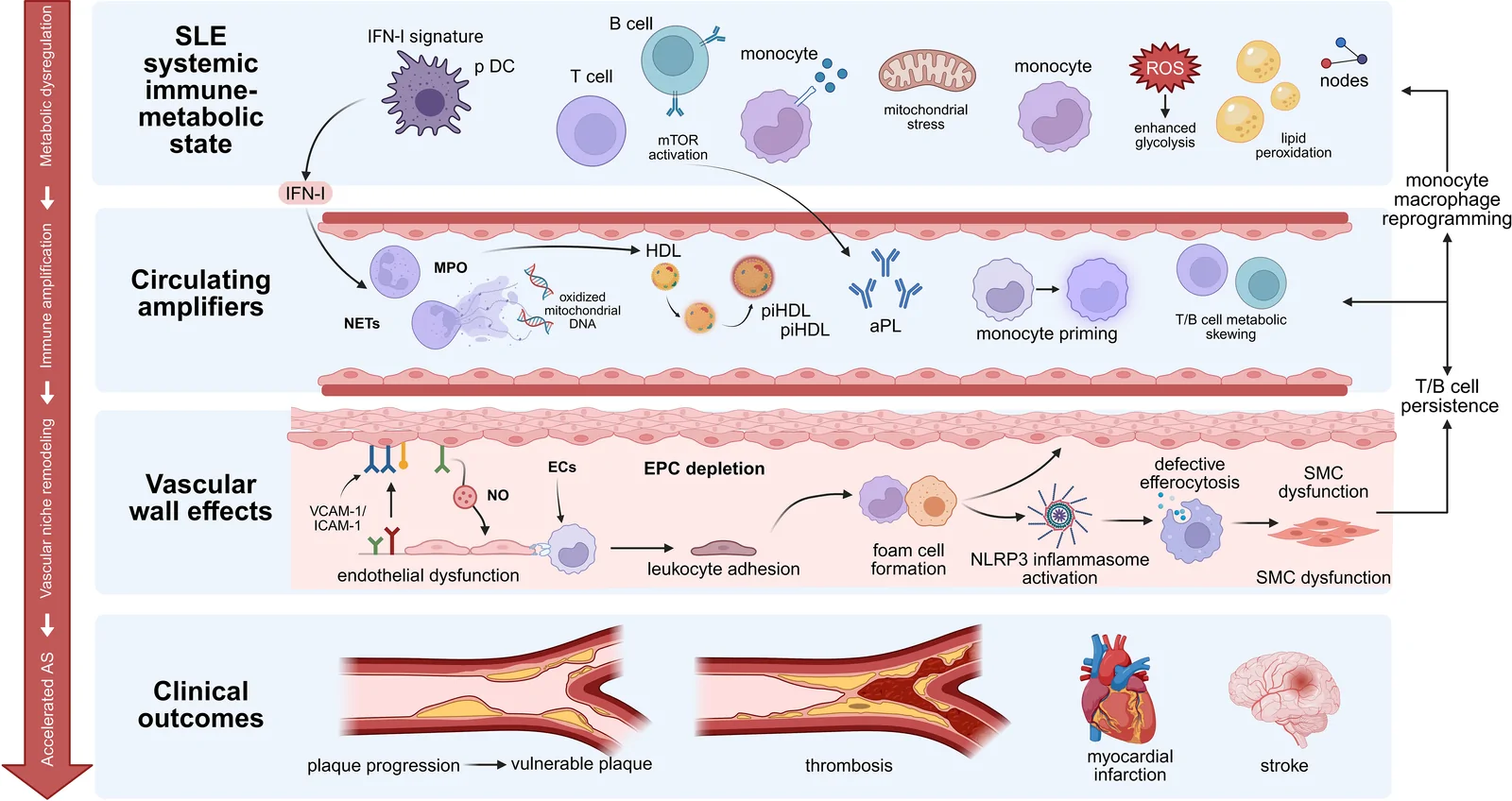
SLE-driven accelerated atherosclerosis: an immune-metabolic-vascular cascade. This schematic summarizes the major stages through which systemic lupus erythematosus (SLE) may accelerate atherosclerosis. The top tier incorporates multi-omics and bioinformatics insights, illustrating how a systemic immune-metabolic state—characterized by enhanced glycolysis, mitochondrial stress, and key interacting nodes—primes the immune system. The cascade then proceeds through: (1) amplification by circulating type I interferon (IFN-I), neutrophil extracellular traps (NETs), and the formation of pro-inflammatory high-density lipoprotein (piHDL); (2) vascular niche remodeling driven by endothelial dysfunction, foam-cell propagation, and NLRP3 inflammasome activation; and (3) progression toward vulnerable plaque and accelerated atherosclerotic cardiovascular disease (ASCVD) events, including myocardial infarction and stroke. Abbreviations: ASCVD, atherosclerotic cardiovascular disease; aPL, antiphospholipid antibodies; ECs, endothelial cells; EPC, endothelial progenitor cell; HDL, high-density lipoprotein; ICAM-1, intercellular adhesion molecule 1; IFN-I, type I interferon; MPO, myeloperoxidase; mTOR, mechanistic target of rapamycin; NETs, neutrophil extracellular traps; NO, nitric oxide; pDC, plasmacytoid dendritic cell; piHDL, pro-inflammatory high-density lipoprotein; ROS, reactive oxygen species; SMC, smooth muscle cell; VCAM-1, vascular cell adhesion molecule 1.

Rather than passively coexisting with atherosclerosis (AS), systemic lupus erythematosus (SLE) should be regarded as an active driver—and, in many patients, a major determinant—of premature vascular injury beyond traditional cardiovascular risk factors (69, 70). Accumulating evidence suggests that SLE accelerates vascular disease through a staged immune–metabolic–vascular cascade rather than through a single isolated mechanism. In this framework, endothelial dysfunction and defective vascular repair constitute an early permissive state; chronic NET burden and lipoprotein dysfunction amplify oxidative vascular inflammation; metabolically preconditioned myeloid cells sustain plaque growth and necrotic core formation; and adaptive immune persistence together with immunothrombosis promote plaque vulnerability (42, 71). Metabolic reprogramming is especially important in this sequence because it does not merely accompany inflammation; instead, it lowers the threshold for IFN-I amplification, NETosis, maladaptive lipid handling, and chronic lesional inflammation. Accordingly, the mechanisms discussed below are best understood not as parallel abnormalities, but as temporally connected stages through which SLE progressively reshapes the vascular wall into a pro-atherogenic niche. This perspective is important because it shifts the interpretation of SLE-associated AS from simple coexistence toward a model of disease-associated vascular remodeling. To visually conceptualize this overarching framework, **Figure 1** integrates recent multi-omics perspectives to map the systemic immune-metabolic state onto the staged IFN–NET–myeloid–adaptive immune cascade that progressively reshapes the arterial wall, providing a mechanistic roadmap for the specific pathways detailed below.

### 4.1 Pathway 1: type I interferon and endothelial axis

The earliest vascular consequences of SLE are best captured by the IFN-I–endothelial axis, in which endothelial activation and impaired vascular repair establish a permissive state for subsequent atherogenesis. SLE disease activity, nephritis, and autoantibody positivity correlate significantly with serum type I interferon (IFN-I) levels. IFN-I is increasingly recognized as an important contributor to cardiovascular involvement in SLE (71). Basic and clinical studies have identified several relevant mechanisms. IFN-I directly inhibits the proliferation of endothelial progenitor cells (EPCs). This inhibition depletes their numbers and weakens the repair capacity of the vascular endothelium (72). In addition, IFN-I may disrupt mitochondrial homeostasis in mature vascular endothelial cells. This disruption leads to the uncoupling of endothelial nitric oxide synthase (eNOS). It may also increase superoxide production.

This local metabolic oxidative stress impairs vasodilation functions that depend on nitric oxide (NO). It also upregulates the expression of adhesion molecules like vascular cell adhesion molecule 1 (VCAM-1) and intercellular adhesion molecule 1 (ICAM-1). These changes create a permissive endothelial phenotype characterized by increased leukocyte adhesion and transmigration, facilitating early lesion initiation. Endothelial dysfunction forms a critical initiating link. It connects the systemic immune dysregulation of SLE with early AS lesions. Clinical observations provide preliminary translational support for this pathway (73). Interventions targeting the IFN pathway have been associated with reductions in IFN signatures and selected vascular/inflammatory readouts in SLE; however, whether IFN blockade translates into reduced plaque progression or ASCVD events requires dedicated endpoint studies.

### 4.2 Pathway 2: NETs and pro-inflammatory HDL axis

Once endothelial susceptibility has been established, persistent NET burden together with oxidative modification of HDL can amplify local vascular inflammation and increase oxidative lipid stress within the arterial wall. Multiple studies indicate that NET clearance is impaired in a subset of patients with SLE (74, 75). Neutrophils are highly prone to undergoing NETosis under specific conditions. These include the strong stimulation of autoantibodies and a cytokine microenvironment rich in IFN-I. Furthermore, impaired DNase activity in the serum of these patients exacerbates the problem. This may contribute to persistent NET accumulation within the circulation and vascular compartment. NETs do not only participate in lupus nephritis. They also act as a core mechanism driving vascular injury and immunothrombosis. The structure of NETs is rich in MPO.MPO is a potent oxidative enzyme. It can directly oxidize key functional proteins of HDL, such as apolipoprotein A-I (ApoA-I). This oxidative modification causes fundamental changes in the structure and function of HDL. This modification abrogates its atheroprotective properties, converting HDL into piHDL (76). From the perspective of lipid metabolism, MPO-mediated oxidation can impair macrophage reverse cholesterol transport (RCT).MPO-mediated oxidation can inhibit a key atheroprotective process. It halts the macrophage reverse cholesterol transport (RCT) mediated by ATP-binding cassette transporter A1 (ABCA1). Loss of this protective function may underlie vascular lipid retention and plaque expansion.

In addition, piHDL no longer possesses anti-inflammatory properties. Instead, it promotes the adhesion and migration of monocytes to the vascular endothelium. It induces the activation of platelet-derived growth factor receptor beta (PDGFRβ) in monocytes. This activation triggers the release of inflammatory factors like tumor necrosis factor-alpha (TNF-α). Ultimately, this directly aggravates sterile inflammation in the vascular wall (77). Simultaneously, the components of NETs themselves exhibit strong procoagulant activity. These components include the DNA backbone, histones, and tissue factor. They provide a physical scaffold for blood clots (78). Prospective cohort studies support the clinical relevance of this pathway (79). They identify piHDL as an independent risk factor for carotid plaques in patients with SLE. Its predictive value may exceed that of selected traditional lipid indices in some cohorts. Persistent vascular inflammation may promote plaque growth. It works synergistically with the highly vulnerable thrombotic microenvironment created by NETs. This synergy not only accelerates plaque progression but also greatly increases the risk of rupture.

### 4.3 Pathway 3: monocyte/macrophage immunometabolic reprogramming

At the stage of lesion propagation, metabolically preconditioned monocytes and macrophages entering the arterial wall are poised to sustain foam-cell formation, inflammasome activation, and defective efferocytosis. In SLE, monocytes and macrophages do not just change in number. They also undergo profound immunometabolic reprogramming. Single-cell transcriptomic and functional studies have identified several relevant metabolic abnormalities. Peripheral blood monocytes in SLE show significant metabolic abnormalities. These include enhanced glycolytic flux, increased production of mitochondrial reactive oxygen species (mtROS), and lipid metabolism disorders (9, 80). The IFN-I signaling pathway directly drives this metabolic phenotype. Its downstream effector molecule is ubiquitin specific peptidase 18 (USP18). USP18 maintains high expression in monocytes. This continuously amplifies glycolytic activity and promotes NLRP3 inflammasome activation. This may establish a positive feedback loop linking IFN signaling, USP18 expression, metabolic remodeling, and inflammatory activation (81). In essence, inflammatory factors like TNF-α and IFN-I systematically drive this pre-reprogramming. This process acts as a pathological form of trained immunity. Monocytes are locked into a pro-inflammatory memory even before entering the vascular wall. Epigenetic modifications and metabolic rewiring cause this effect. This memory features high glycolysis, high ROS levels, and classic M1 polarization (82, 83).

The SLE inflammatory milieu may precondition monocytes and macrophages before vascular entry. Their pathological behaviors are then significantly amplified upon entering the arterial intima: (1) Impaired lipid handling: On the lipid metabolism side, IFN-I signaling significantly inhibits the liver X receptor (LXR) in macrophages. It also suppresses its downstream transcriptional targets. These targets include ABCA1 and ATP binding cassette subfamily G member 1 (ABCG1). This suggests that SLE-associated macrophages may display increased scavenger receptor-mediated uptake of oxLDL, cellular cholesterol efflux pathways are significantly suppressed. Together, these changes may promote foam-cell formation (10). (2) Inflammatory cascade: Elevated mtROS levels and a reliance on glycolysis enhance NLRP3 inflammasome activation. This promotes the excessive release of cytokines like IL-1β and IL-18 (10). (3) Necrotic core expansion: The efferocytosis capacity of macrophages is severely impaired. This leads to a failure in clearing apoptotic cells within the plaque. It subsequently causes the fibrous cap to thin and increases plaque vulnerability. Human metabolomics studies also confirm this clinical link. Specific lipidomes and energy metabolism small molecule profiles in SLE plasma correlate significantly with the degree of coronary artery calcification (CAC) (84). Monocyte and macrophage metabolic reprogramming may therefore serve as an important mechanistic link between systemic immune dysregulation in SLE and local AS progression.

An emerging concept that further strengthens this model is that lesional metabolism is not merely a passive reflection of systemic inflammation, but is locally organized within the plaque microenvironment (85). Recent single-cell and spatial omics studies in human atherosclerotic tissue have identified anatomically restricted endothelial, myeloid, and stromal niches with distinct inflammatory and metabolic programs, including proangiogenic endothelial states in advanced lesions and specialized macrophage subsets occupying different plaque regions (86). These findings suggest that once preconditioned SLE monocytes enter the arterial wall, they are not simply exposed to a generic inflammatory milieu; rather, they may be incorporated into spatially structured immunometabolic niches that reinforce glycolytic bias, oxidative stress, defective efferocytosis, and tissue remodeling (87). From this perspective, systemic lupus does not only increase the influx of pathogenic cells into the plaque, but may also bias how those cells are metabolically instructed and retained within the lesional microenvironment.

The emerging paradigm of clonal hematopoiesis of indeterminate potential (CHIP) may further refine the understanding of myeloid-driven atherogenesis. Somatic mutations within hematopoietic stem cells, predominantly in epigenetic regulators such as TET2, can generate clonally expanded myeloid populations with enhanced inflammatory potential. Specifically, TET2-mutant macrophages demonstrate aberrant hyperactivation of the NLRP3 inflammasome, promoting IL-1β secretion and potentially accelerating atherogenesis (88). In the context of autoimmune diatheses like SLE, chronic systemic inflammation is postulated to exert selective pressure, prematurely driving the expansion of these mutant hematopoietic clones. Thus, interactions between CHIP-associated myeloid dysfunction and the SLE inflammatory microenvironment may create a feed-forward inflammatory loop that amplifies IL-1β-related cardiovascular risk (89). However, direct evidence linking CHIP to cardiovascular outcomes specifically in SLE remains limited, and this interaction should be regarded as an emerging hypothesis.

### 4.4 Pathway 4: T- and B-cell metabolic abnormalities, arterial wall seeding, and persistence

In more advanced lesions, metabolically dysregulated T- and B-cell responses, together with autoantibody burden and immunothrombotic tendency, may further sustain local inflammation, weaken plaque stability, and increase the likelihood of thrombotic complications. Lymphocytes experience continuous activation and significant metabolic reprogramming in systemic lupus erythematosus (SLE). Researchers believe this immunometabolic abnormality drives systemic inflammation. It also likely creates favorable conditions for chronic immune responses directly within the vascular wall.

#### 4.4.1 T cell metabolic imbalance and colonization

CD4^+^ T cells in SLE exhibit continuous activation of the mTORC1 signaling pathway. Their core metabolic feature is significantly enhanced aerobic glycolysis (29). Although their mitochondria show membrane hyperpolarization (MHP), the energy production capacity of oxidative phosphorylation (OXPHOS) is actually impaired. This leads to a relative depletion of ATP and a massive accumulation of ROS. This abnormal metabolic program drives the differentiation of pro-inflammatory Th1 and Th17 cells. It also promotes the expansion of specific double-negative (DN) T cells. At the same time, it inhibits the generation of protective Treg cells, which rely on fatty acid oxidation (FAO) and normal OXPHOS. This Th17/Treg disequilibrium may weaken physiological regulatory mechanisms that normally constrain vascular inflammation. In an environment with long-term high expression of IFN-I, the vascular endothelium upregulates chemokines like C-X-C motif chemokine ligand 9 (CXCL9) and CXCL10. This selectively recruits CXCR3^+^ and CCR5^+^ pro-inflammatory CD4^+^ T cells, which may possess metabolic features that favor recruitment to the arterial wall (90). Multiple prospective clinical studies have confirmed this link. The levels of CCR5^+^ CD4^+^ T cells in the peripheral blood of patients with SLE independently correlate with the occurrence of carotid AS (91). T cells do not merely act as transient inflammatory infiltrating cells after entering the plaque. Single-cell transcriptomic and T-cell receptor (TCR) sequencing studies suggest a more persistent local T-cell presence within human AS plaques. Clonally expanded CD4^+^ and CD8^+^ T cell populations exist within human AS plaques. They display transcriptional features related to continuous activation and tissue residency, such as CD69 expression. This suggests they may survive locally for long periods and receive antigen-driven stimulation (92). We cannot entirely equate them with the classical definition of tissue-resident memory T (Trm) cells. However, these cells do show molecular features similar to tissue-resident-like immune responses. Functionally, T cells within the plaque predominantly exhibit a Th1-related phenotype. They continuously produce IFN-γ, and some studies have also detected IL-17 expression (54). Researchers have proven that IFN-γ can inhibit collagen synthesis in vascular smooth muscle cells and weaken their repair capacity. Simultaneously, it promotes the polarization of macrophages toward an inflammatory phenotype (93). Therefore, the continuous activation of T cells driven by local antigens likely impacts smooth muscle cell repair and myeloid cell polarization. Together, these processes maintain the inflammatory microenvironment within the plaque and promote lesion progression.

In addition to mTOR-driven hyperactivation during active disease, chronic SLE may involve exhausted-like or senescent T-cell states characterized by metabolic maladaptation. Sustained autoantigenic exposure attenuates their glycolytic capacity, precipitating severe mitochondrial perturbations—specifically, ultrastructural fragmentation, transmembrane potential depolarization, and excessive mtROS generation. Adaptation to the hypoxic and oligotrophic microenvironment of the atherosclerotic plaque necessitates a compensatory reliance on aberrant lipid metabolism and fatty acid oxidation (FAO). Consequently, this persistent immunometabolic stress induces a senescence-associated secretory phenotype (SASP), continuously propagating vascular endothelial injury and accelerating atherogenesis (94).

#### 4.4.2 B cell and autoantibody axis

In terms of humoral immunity, B cells in SLE also exhibit enhanced PI3K-Akt-mTOR signaling and metabolic reprogramming features. This state supports the continuous differentiation and survival of effector B cells and plasma cells (95). Metabolism-driven humoral immune activation relates closely to the production of multiple autoantibodies. These include antiphospholipid antibodies (aPL). aPL are widely recognized as important risk factors for arterial thrombosis and myocardial infarction (96). Studies have suggested an enhanced β2-glycoprotein I (β2-GPI)-specific T cell response in patients with SLE who have secondary antiphospholipid syndrome (APS). This response also shows a Th1 bias (97). Evidence for the systematic isolation and identification of β2-GPI-specific Th17 clones directly within human atherosclerotic plaques remains relatively limited. However, existing data support the idea that autoantibodies and autoantigen-specific T cells likely participate in local vascular inflammatory responses. In the context of SLE, these findings support a plausible mechanism in which metabolism-driven humoral immune activation provides a sustained source of antigenic stimulation. The continuous generation of autoantibodies, driven by B cell metabolism, may provide an ongoing source of antigen stimulation. This may sustain autoantigen-driven T- and B-cell interactions within the vascular wall. Consequently, some atherosclerotic plaques exhibit immunological features that more closely resemble an autoantigen-driven chronic inflammatory state.

### 4.5 Emerging modifiers: viral immunometabolic stress and vascular injury

Chronic or latent viral reactivation, particularly cytomegalovirus (CMV) and Epstein-Barr virus (EBV), may act as an emerging modifier of SLE-accelerated atherosclerosis (98). Rather than merely acting as independent, generic cardiovascular risk factors, these viral agents specifically synergize with the core SLE pathophysiological network.

In the context of SLE—where systemic tolerance is breached and patients are frequently subjected to immunosuppressive regimens—latent viruses may have increased opportunities for reactivation. Viral nucleic acids serve as potent exogenous pathogen-associated molecular patterns (PAMPs) that chronically engage endosomal Toll-like receptors (TLR7/9) and cytosolic sensors (e.g., cGAS-STING) within plasmacytoid dendritic cells (pDCs) and myeloid compartments (99, 100). Importantly, persistent viral sensing may not represent a fully independent disease process; instead, it directly fuels the pre-existing SLE autoantigen-driven IFN-I amplification loop. This exogenous viral stimulus compounds the endogenous nucleic acid burden (such as circulating mtDNA and NETs), thereby potentially lowering the threshold for systemic IFN-I overproduction and sustaining the interferon signature (101).

From an immunometabolic perspective, this superimposed viral signaling further entrenches the aberrant glycolytic reprogramming of macrophages and exacerbates IFN-mediated endothelial progenitor cell (EPC) depletion. Consequently, viral triggers may function as disease-contextual amplifiers of the IFN-I-driven immune–metabolic–vascular cascade and accelerating atherogenesis, particularly in highly immunosuppressed or IFN-high SLE endotypes (102).

### 4.6 Multi-omics evidence supporting the immune-metabolic-vascular axis

High-resolution profiling using single-cell RNA sequencing (scRNA-seq) and integrated multi-omics provides supportive evidence for the proposed immune-metabolic-vascular cascade. At the systemic level, transcriptomic characterization of peripheral blood mononuclear cells (PBMCs) from patients with SLE—specifically monocytes and T/B lymphocytes—has identified pre-activated immune signatures. Prior to tissue infiltration, these circulating subsets exhibit a pronounced type I interferon (IFN-I) response, enhanced glycolytic flux, profound mitochondrial perturbations, and elevated oxidative stress (103).

Concurrently, at the lesional level, scRNA-seq and spatial omics of human atherosclerotic plaques have unveiled a highly structured microenvironment. Within the arterial wall, endothelial cells, macrophages, T cells, and stromal cells do not merely coexist but may form spatially restricted inflammatory niches with distinct transcriptomic and metabolic programs (92).

Integrating these systemic and lesional datasets elucidates a critical bridging mechanism. When the metabolically preconditioned immune cells of patients with SLE infiltrate the arterial intima, their pre-existing inflammatory and metabolic bias may favor retention and further instruction within these plaque niches. This spatial retention may interact with their pre-existing metabolic bias to promote macrophage foam-cell formation,NLRP3 inflammasome hyperactivation, and the perpetuation of localized vascular immunity (30, 87, 104, 105). To systematically synthesize these rapidly evolving insights, a comprehensive overview mapping these systemic metabolic aberrations to localized vascular injury across different omics modalities is provided in **Table 1**. However, these findings should be interpreted with appropriate clinical caution. Metabolic dysregulation should be considered an upstream amplifier rather than a universal initiating cause in all patients with SLE. Rather than acting in isolation, this metabolic rewiring functions collaboratively with autoantigenic and genetic factors to propel the immune-metabolic-vascular disease continuum.

**Table 1:** Multi-omics evidence supporting metabolic dysregulation as an upstream amplifier in SLE-associated atherosclerosis.

Omics modality	Biospecimen or target	Key metabolic aberrations	Linked inflammatory pathways	Contribution to atherogenesis	Current evidence limitations
Bulk transcriptomics	PBMCs, monocytes, T and B cells	Enhanced glycolytic pathway enrichment and IFN-associated metabolic rewiring	IFN-I–JAK–STAT axis	Primes circulating monocytes and lymphocytes toward hyper-reactive inflammatory phenotypes before vascular entry.	Largely based on surrogate biomarkers and pathway-enrichment analyses rather than hard cardiovascular endpoints.
Single-cell and spatial omics	Monocytes, macrophages, endothelial cells, and plaque-resident immune cells	Expansion of IFN-responsive and pro-inflammatory myeloid subsets with glycolytic and oxidative stress programs	IFN-responsive myeloid inflammation; NLRP3–IL-1β axis	May promote foam-cell formation, endothelial activation, and maintenance of the lesional inflammatory niche.	Direct single-cell or spatial datasets from SLE-specific atherosclerotic plaques remain scarce.
Metabolomics	Plasma and serum	Altered lipid metabolism, accumulation of oxidized lipid species, and mitochondrial stress-related metabolites	ROS-driven NETosis and lipid peroxidation	Associated with coronary artery calcification (CAC), oxidative vascular injury, and subclinical atherosclerosis (AS).	Primarily associative; direct causal mechanisms in human SLE require further validation.
HDL lipidomics and functional profiling	HDL particles	HDL remodeling, reduced PON1 activity, impaired antioxidant capacity, and defective cholesterol efflux	NET-derived MPO-mediated HDL oxidation	Drives pro-inflammatory HDL (piHDL) formation and impairs macrophage cholesterol efflux, favoring foam-cell accumulation.	Often limited by small cohort sizes, cross-sectional designs, and heterogeneous HDL functional assays.

AS, atherosclerosis; CAC, coronary artery calcification; HDL, high-density lipoprotein; IFN-I, type I interferon; IL-1β, interleukin-1 beta; JAK-STAT, Janus kinase-signal transducer and activator of transcription; MPO, myeloperoxidase; NETs, neutrophil extracellular traps; NLRP3, NLR family pyrin domain containing 3; PBMCs, peripheral blood mononuclear cells; piHDL, pro-inflammatory high-density lipoprotein; PON1, paraoxonase-1; ROS, reactive oxygen species; scRNA-seq, single-cell RNA sequencing; SLE, systemic lupus erythematosus.

## 5 Therapeutic outlook: a layered framework integrating immunometabolic, immune-specific, and lipid/vascular-wall strategies

Atherogenesis in SLE extends beyond conventional hypercholesterolemia, presenting as a multi-axis pathology shaped by continuous immune activation, immunometabolic reprogramming, and inherent vascular vulnerability (10, 106). Therefore, the therapeutic strategy should not simply mean adding an immunosuppressive drug to routine lipid-lowering regimens. Treatments must instead be stratified and integrated around the causal chain established earlier. This specific chain involves the amplification of IFN-I and NETs, metabolic skewing in myeloid and lymphoid cells, lipoprotein dysfunction, and an imbalanced plaque inflammatory microenvironment. The current levels of evidence vary substantially across therapeutic strategies. Some interventions only find support in animal models or *in vitro* mechanisms. Other approaches have successfully improved disease activity or biomarkers in human SLE patients. However, dedicated studies utilizing plaque imaging or hard atherosclerotic cardiovascular disease (ASCVD) events as primary endpoints remain quite limited (107, 108).

Furthermore, achieving systemic remission stands as a necessary prerequisite. Yet, this systemic control often fails to fully halt the progression of vascular lesions. SLE-related immune activation may initiate vascular susceptibility, while immunometabolic reprogramming may amplify lesion progression. This biological dynamic prevents simple monotherapies targeting isolated lipid loads or general inflammation from breaking the self-sustaining cycle of SLE-AS (109, 110). Consequently, translating this immune-metabolic-vascular framework into clinical practice demands a paradigm shift in therapeutic design. Future therapeutic development may therefore require a shift from single-target control toward stratified, mechanism-guided combination strategies. Based on this rationale, this chapter introduces a three-dimensional defense framework that aligns with the specific stages of disease progression. The first dimension involves upstream metabolic resetting. This approach aims to attenuate the initial systemic inflammatory drivers directly at their cellular origin. The second dimension relies on midstream immune-specific blockade. This strategy abrogates pathogenic signaling cascades driven by interferons, B cells, and autoantibodies. The final dimension provides downstream lipid and vascular wall protection. This layer aims to reduce vascular vulnerability to lipid oxidation, inflammasome activation, and immunothrombosis. Given the current evidence base, researchers and clinicians should view this framework as a mechanistic roadmap. It effectively organizes existing therapeutic evidence and guides future trial designs, rather than representing a clinical standard of care already validated by hard endpoints (111, 112). Accordingly, this framework should be interpreted as a mechanism-guided roadmap layered on top of standard SLE care and conventional cardiovascular prevention, rather than as a substitute for either (108).

### 5.1 The upstream hub layer: immunometabolic resetting rather than direct plaque reversal

Upstream strategies aim to attenuate shared immunometabolic amplifiers of SLE-related AS. This amplifier is the immunometabolic bias mediated by the mTOR–AMPK–ROS axis (10). Current evidence shows that mTOR inhibition, redox balance restoration, or metabolic regulation can indeed improve disease activity and immune phenotypes in human patients with SLE. However, this evidence primarily reflects lupus activity and immunometabolic readouts. It does not automatically equate to a direct improvement in vascular lesions.

A single-arm, open-label phase I/II study evaluated sirolimus (rapamycin) in patients with active SLE. The treatment was associated with reduced overall disease activity. It also corrected the imbalance within the pro-inflammatory T cell lineage. These clinical findings strongly support mTOR as a druggable immunometabolic hub (27). Furthermore, a randomized double-blind trial of NAC yielded positive results. It suggested that NAC can improve SLE disease activity by inhibiting mTOR activation in T cells (26). Metformin demonstrates a clear metabolic correcting effect in lupus-susceptible animal models (29). However, its clinical translation faces distinct challenges. A randomized double-blind trial investigated metformin in non-diabetic patients with SLE. The primary endpoint regarding disease flares did not reach overall statistical significance. The authors explicitly noted that the study was underpowered (113). Overall, interventions at this upstream layer have shown evidence of improving disease activity or immunometabolic phenotypes in selected SLE cohorts. Nevertheless, their anti-atherosclerotic effects currently rely mostly on mechanistic extrapolation. There is still a distinct lack of definitive evidence regarding hard vascular outcomes (113).

### 5.2 Midstream immune-specific blockade: reducing SLE-associated immune pressure on the vascular wall

The midstream layer targets SLE-specific immune pathways that may impose sustained inflammatory pressure on the vascular wall. It specifically aims to reduce the continuous immune pressure exerted by SLE-associated pathways on the vascular wall. Key targets include the IFN-I signaling axis alongside the B cell, autoantibody, and immune complex pathways (106). This layer addresses SLE-specific pathogenic mechanisms more directly than the upstream hub layer. However, clinical evidence for potential vascular benefits currently remains confined to biomarkers and surrogate endpoints (108).

#### 5.2.1 JAK-STAT axis intervention: tofacitinib

A randomized, double-blind, placebo-controlled phase I trial evaluated tofacitinib in SLE. The drug improved several immune and cardiometabolic readouts associated with premature atherosclerosis in the short term. These specific readouts included IFN-related transcriptional signals, NET indices, and certain vascular function parameters (114). This suggests that blocking the JAK-STAT pathway can simultaneously affect the IFN axis and vascular metrics in humans. However, this study had a small sample size. Its primary endpoint also remained strictly focused on safety. More importantly, broader safety concerns exist. The ORAL Surveillance study investigated JAK inhibitors in high-risk patients with rheumatoid arthritis. Both this study and safety warnings from the Food and Drug Administration (FDA) indicate potential dangers. These agents have been associated with increased risks of cardiovascular events, thrombosis, and malignancies in selected high-risk populations (115). Therefore, researchers must perform independent risk stratification before testing JAK inhibitors as a preventive intervention for SLE-related atherosclerosis. Future proof-of-concept studies should prioritize the exclusion of specific high-risk endotypes. These include patients positive for antiphospholipid antibodies (aPL) and those with antiphospholipid syndrome (APS). Individuals with other high-thrombosis risk profiles must also be excluded. These patients already have an increased baseline risk of arterial and venous thrombosis. Their underlying conditions could further amplify the safety concerns associated with JAK inhibitors (116). Based on current evidence, JAK inhibitors are not suitable as a routine vascular protective regimen for SLE-AS. Instead, they represent a candidate strategy. Their potential use requires careful endotype selection, strict thrombosis risk assessment, and long-term outcome validation.

#### 5.2.2 IFNAR blockade: anifrolumab

Anifrolumab is a monoclonal antibody targeting IFNAR1.It improved disease activity in phase III trials involving patients with active SLE such as TULIP-2. These results successfully demonstrate the clinical druggability of the IFN-I axis (117). However, the existing evidence primarily relies on disease activity endpoints. It lacks data regarding subclinical plaques, vascular imaging, or ASCVD events. Intervention targeting the IFN axis is now firmly established in routine clinical SLE management. Yet, the field still lacks dedicated studies utilizing plaque or cardiovascular event endpoints. Therefore, it remains unclear whether this treatment can truly alter the natural history of SLE-related AS.

#### 5.2.3 B-cell activating factor blockade: belimumab

Belimumab represents the B cell-specific therapeutic pathway. Phase III trials such as BLISS-52 and BLISS-76 have firmly established its clinical efficacy. The drug effectively reduces SLE disease activity and overall immune burden (118, 119). Recent studies have also reported vascular-relevant surrogate findings. High-density lipoprotein (HDL) demonstrates improved cholesterol efflux capacity and antioxidant function following belimumab treatment. Paraoxonase-1 (PON1) activity also increases. These functional gains occur alongside distinct improvements in HDL lipidomic profiles (120). This observation provides mechanistic support for a potential link between B-cell-targeted therapy and lipoprotein function. It suggests that B cell-targeted therapy might indirectly correct qualitative dysfunctions in lipoproteins. However, these specific metrics still represent surrogate endpoints. Belimumab is already a mature targeted therapy for SLE. It shows clear potential to improve HDL functional readouts related to AS. Yet, this evidence primarily remains at the surrogate endpoint level. An important evidence gap remains. Researchers must conduct further studies to prove its true ability to reduce actual plaque burden or hard cardiovascular events.

#### 5.2.4 CD19 CAR-T cell therapy: a paradigm shift in B-cell depletion

CD19 CAR T-cell therapy has emerged as a potentially transformative strategy for refractory SLE. Unlike conventional biologics, which may incompletely deplete autoreactive B-cell compartments, CAR T-cells induce a profound “immunological reset” by eradicating pathogenic B-cell clones.

A landmark investigation by Mackensen et al. (121) demonstrated that a single infusion induced rapid, treatment-free clinical remission in patients with severe refractory SLE, characterized by autoantibody seroconversion and complement normalization. Subsequent follow-up data (2024-2025) corroborate the long-term durability of this intervention, revealing that the emergent B-cell repertoire post-reconstitution maintains a “naive,” non-autoreactive phenotype. From an immunometabolic perspective, deep B-cell depletion could theoretically attenuate upstream drivers of accelerated atherosclerosis by terminating perpetual pro-atherogenic autoantibody generation and IFN-I amplification loop (122). Nevertheless, empirical evidence substantiating a cardiovascular protective effect remains absent. Current trials lack data on surrogate vascular metrics (e.g., CIMT) or hard ASCVD outcomes. Furthermore, high cost, lymphodepleting preconditioning, and potentially severe toxicities—namely cytokine release syndrome (CRS) and immune effector cell-associated neurotoxicity syndrome (ICANS)—preclude its broad prophylactic application in unselected SLE cohort (123). Thus, while CAR T-cell therapy offers durable SLE remission, its utility for mitigating SLE-accelerated atherosclerosis remains speculative, necessitating prospective trials integrating cardiometabolic biomarkers.

### 5.3 Downstream lipid and plaque microenvironment layer: vascular wall protection beyond low-density lipoprotein cholesterol

Upstream and midstream strategies primarily aim to weaken systemic immune drivers. The downstream layer focuses more directly on the plaque microenvironment and clinical event risk. It directly targets the local plaque microenvironment and the risk of clinical events. LDL-C lowering alone may be insufficient in SLE-associated AS. This singular approach cannot fully explain or reverse the residual cardiovascular risk. The LAPS focused on adult patients with SLE. The Atherosclerosis Prevention in Pediatric Lupus Erythematosus (APPLE) trial investigated pediatric and adolescent patients. Both trials showed that atorvastatin effectively improves lipid profiles. However, they generally failed to reach statistical significance for primary subclinical atherosclerosis endpoints (124) (125). This observation does not negate the routine value of statins for patients with SLE who meet general indications for atherosclerotic cardiovascular disease (ASCVD).Instead, it highlights an important limitation of lipid-centered prevention in SLE. Vascular risk in SLE is not solely driven by LDL-C. Simple lipid-lowering therapy cannot adequately address the residual risk caused by immune-driven inflammation and qualitative lipoprotein dysfunction.

#### 5.3.1 Intensive lipid-lowering and potential inflammatory synergy: proprotein convertase subtilisin/kexin type 9 inhibitors

Against this background, PCSK9 inhibitors are supported by strong cardiovascular outcome evidence in general ASCVD populations. However, their application in SLE still remains an extrapolation. The FOURIER trial evaluated evolocumab. The ODYSSEY OUTCOMES trial investigated alirocumab. Both trials focused on general ASCVD populations. They demonstrated that these drugs can further reduce major adverse cardiovascular events (MACE) when added to baseline statin therapy (126, 127). Meanwhile, preclinical studies suggest a broader role for PCSK9. It may help regulate macrophage scavenger receptors and inflammatory responses related to oxidized low-density lipoprotein (oxLDL). This dual action offers a potential lipid-inflammation synergistic target for immune-driven AS (128). However, an important limitation should be noted. This mechanistic evidence still stems primarily from animal models or *in vitro* experiments. Dedicated studies evaluating the effects of PCSK9 inhibitors on plaque progression or cardiovascular events specifically in SLE are currently lacking. At this stage, physicians can consider PCSK9 inhibitors as an intensive lipid-lowering option for very high-risk patients with SLE. These candidates include individuals with a prior history of ASCVD or persistently elevated LDL-C levels. This clinical approach simply follows general ASCVD management frameworks. Nevertheless, the incremental benefits of these drugs against SLE-specific residual inflammatory risk remain unproven. Future imaging and mechanistic biomarker studies must further validate these potential advantages.

#### 5.3.2 Regulation of the plaque inflammatory microenvironment: the interleukin-1 beta and NLR family pyrin domain containing 3 axis alongside colchicine

Non-lipid inflammatory mechanisms are also important in plaque progression. Sterile inflammation within the plaque plays a central role. Inflammasome-driven microenvironmental imbalances also significantly contribute to this pathological process. Cholesterol crystals and oxidized lipids can activate the NLRP3 inflammasome. This activation subsequently promotes the release of IL-1β. The CANTOS trial focused on a general ASCVD population. It demonstrated that targeting IL-1β with canakinumab reduces MACE. This clinical benefit occurred without any additional lowering of LDL-C. This trial provided a clinically important example of event reduction through targeted anti-inflammatory therapy (129). Similarly, low-dose colchicine reduced cardiovascular event risks in the COLCOT and LoDoCo2 trials. These trials involved patients with chronic coronary disease. These results suggest that neutrophil- and inflammasome-related plaque inflammation is therapeutically modifiable in non-SLE coronary populations (130, 131).

While these landmark trials confirm that the lesional inflammatory microenvironment is highly modifiable, extrapolating IL-1β-targeted and inflammasome-modulating therapies to the SLE population necessitates rigorous risk-benefit stratification. In the CANTOS trial, the atheroprotective benefits of canakinumab were significantly counterbalanced by an elevated incidence of fatal infections. This is particularly relevant for patients with SLE, who inherently exhibit compromised antimicrobial defenses invariably compounded by the chronic administration of glucocorticoids and broad-spectrum immunosuppressants (132). Consequently, the additive pharmacological blockade of the IL-1β axis could increase susceptibility to severe or opportunistic infections. Therefore, these interventions cannot be routinely implemented for SLE-associated cardiovascular prevention at present. Rather, they should be strictly viewed as candidate pathways reserved for carefully delineated endotypes—specifically those characterized by hyperactive inflammasome signatures, heavy NET burden, or profound immunothrombotic tendencies (30). Future prospective validation must integrate surrogate vascular endpoints with stringent infectious disease surveillance to ensure therapeutic safety.

#### 5.3.3 Bridging systemic and local pathways: novel cardiometabolic drugs (SGLT2i and GLP-1 RAs)

Novel cardiometabolic drugs may exert cardiovascular benefit through systemic metabolic reprogramming (133, 134)—with fasting-mimicry-like effects particularly described for SGLT2 inhibitors—while also directly modulating endothelial homeostasis and the local plaque inflammatory microenvironment (135). In this sense, these agents are distinct from conventional lipid-lowering or anti-inflammatory therapies, because they potentially act at both the systemic and lesional levels. However, the current evidence remains in its early stages.SGLT2 inhibitors have demonstrated cardiorenal benefits in patients with diabetes, heart failure, and chronic kidney disease. GLP-1 receptor agonists exhibit clear cardiovascular protective signals in general metabolic and obese populations. For example, the SELECT trial showed that semaglutide reduced MACE in overweight or obese patients with preexisting cardiovascular disease (136). Preclinical and translational studies provide additional mechanistic insights. Both classes of drugs may influence the atherosclerotic process. They can reduce oxidative stress and improve endothelial function. They also regulate macrophage inflammatory states and inhibit inflammasome-related signaling. Evidence regarding SGLT2 inhibitors is relatively more direct concerning macrophage inflammasome activation, oxidative stress, and metabolic skewing. GLP-1 receptor agonists are more closely associated with endothelial protection, antioxidant effects, and broad inflammatory regulation (135, 137). Therefore, these mechanisms partially overlap with the upstream immunometabolic hub. However, their current clinical relevance stems primarily from cardiorenal and cardiovascular outcome data. Consequently, this chapter more appropriately classifies them within the downstream protection layer.

Within the SLE population, existing evidence primarily focuses on cardiorenal and metabolic outcomes. It lacks data on vascular imaging, plaque phenotypes, or ASCVD event endpoints. More importantly, the strongest human evidence currently stems from a specific clinical subgroup. This subgroup consists of patients with SLE and comorbid type 2 diabetes (T2D). The data do not reflect the broader, unstratified SLE population. Regarding SGLT2i, observational studies and target trial emulations suggest a specific clinical association. These drugs correlate with lower risks of lupus nephritis, heart failure, and all-cause mortality (138, 139). For GLP-1 RAs, small retrospective cohorts indicate generally acceptable tolerability. Newer real-world studies provide further insights. They suggest an association with lower risks of adverse cardiorenal outcomes and mortality specifically within the SLE and T2D subgroup (140, 141). Therefore, these two drug classes may be considered emerging candidate strategies. They possess a preliminary foundation for human clinical translation. However, they are not yet proven vascular protective regimens. Their true impact on subclinical atherosclerosis, lipoprotein function, and ASCVD events remains unclear. Researchers must further clarify these clinical effects through dedicated prospective studies. These future trials need to include broader, unstratified SLE populations.

### 5.4 Summary: therapeutic prospects require evidence-based stratification over empirical polypharmacy

**Figure 2:**
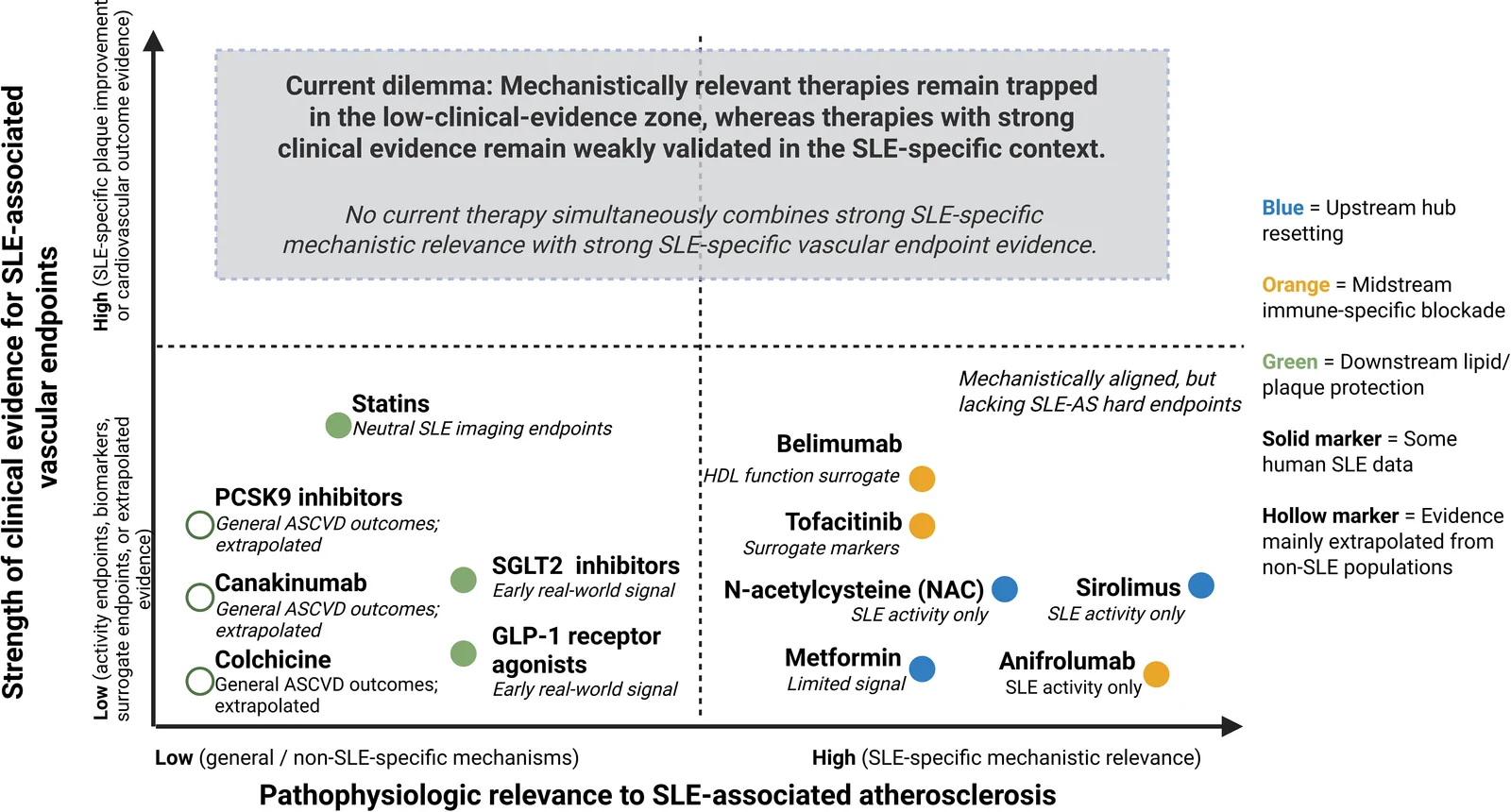
Evidence–mechanism matrix of candidate therapies for SLE-associated atherosclerosis. This conceptual matrix positions candidate pharmacologic interventions according to their pathophysiologic relevance to SLE-associated atherosclerosis (x-axis) and the strength of clinical evidence for SLE-relevant vascular endpoints (y-axis). The figure highlights the current translational gap: therapies most closely aligned with the SLE-AS disease axis are supported mainly by biomarker, disease-activity, or surrogate-endpoint data, whereas therapies with the strongest cardiovascular outcome evidence remain largely extrapolated from general or non-SLE populations. Colors denote therapeutic layer, with blue indicating upstream hub resetting, orange midstream immune-specific blockade, and green downstream lipid/plaque protection. Solid markers indicate therapies supported by some human SLE data, whereas hollow markers indicate therapies whose major cardiovascular evidence derives primarily from non-SLE populations. Abbreviations: ASCVD, atherosclerotic cardiovascular disease; GLP-1, glucagon-like peptide-1; NAC, N-acetylcysteine; PCSK9, proprotein convertase subtilisin/kexin type 9; SGLT2, sodium-glucose cotransporter-2; SLE, systemic lupus erythematosus.

Overall, no standard SLE-specific regimen has been established for preventing or attenuating SLE-related ASCVD events. No dedicated studies have proven a direct reduction in SLE-related ASCVD events. Existing strategies remain biologically and clinically relevant. They target distinct pathological nodes of SLE-AS. However, they operate across vastly different levels of evidence. Upstream immunometabolic resetting primarily improves disease activity in humans. It also enhances various immunometabolic readouts. Midstream blockade targets the IFN and BAFF axes. Human randomized trials prove that these interventions improve lupus activity. They also positively affect certain vascular-related biomarkers. Nevertheless, these studies still lack hard plaque or event endpoints. Downstream strategies address lipids and plaque inflammation. These approaches are supported by the strongest outcome evidence in general ASCVD populations. Yet, researchers have not specifically validated them within SLE cohorts. Future research should not merely expand the number of candidate interventions. Investigators should instead focus on high-risk endotypes. These include patients with high IFN signatures, heavy NET burdens, high levels or piHDL, or aPL/APS positivity. Researchers must systematically link mechanistic biomarkers with surrogate vascular imaging endpoints. They must then connect these metrics to ultimate ASCVD event outcomes. Such an approach will help determine which interventions can control lupus activity while also modifying the trajectory of SLE-associated vascular disease. This translational mismatch is summarized in **Figure 2**, which maps candidate therapies according to their alignment with the SLE-AS disease axis and the strength of SLE-relevant vascular evidence. Complementing this visual matrix, **Table 2** details the specific evidence hierarchy, mechanistic rationale, and clinical positioning for each candidate strategy.

**Table 2:** Evidence hierarchy and translational positioning of therapeutic strategies for SLE-associated atherosclerosis.

Therapeutic category	Representative strategies	Main mechanistic rationale	Evidence status	Translational interpretation
Foundational SLE background therapy	Hydroxychloroquine (HCQ)	Inhibits endosomal TLR7/9 signaling, attenuates IFN-related immune activation, reduces glucocorticoid exposure, and may provide antithrombotic and metabolic benefits	Guideline-supported standard therapy for most patients with SLE; observational and target-trial emulation studies suggest associations with lower cardiovascular and thromboembolic risks, but randomized SLE-AS vascular endpoint trials are lacking	Should be regarded as foundational background therapy for cardiovascular risk modification in SLE, rather than an experimental SLE-AS-specific intervention
Upstream immunometabolic resetting	Sirolimus/rapamycin, N-acetylcysteine (NAC), metformin	Targets mTOR–AMPK–ROS-related metabolic stress, T-cell metabolic overactivation, mitochondrial dysfunction, and redox imbalance	Human SLE data support improvement in disease activity or immunometabolic readouts, but dedicated plaque imaging or ASCVD outcome evidence is lacking	Mechanistically relevant, but vascular benefit remains inferential and requires endpoint-driven validation
IFN–JAK axis blockade	Tofacitinib/JAK inhibitors, anifrolumab	Suppresses IFN-related transcriptional programs, JAK–STAT signaling, NET-related signals, and endothelial inflammatory activation	Anifrolumab has phase III SLE activity evidence; tofacitinib has small phase I data with immune and cardiometabolic readouts; vascular endpoint evidence remains limited	Strong SLE-mechanistic alignment, but not validated as routine SLE-AS prevention; JAK inhibitors require careful thrombotic and cardiovascular risk stratification
B-cell-targeted immune modulation	Belimumab	Inhibits BAFF, reduces B-cell immune burden and autoantibody-related inflammation, and may improve HDL cholesterol efflux and antioxidant function	Phase III trials support SLE efficacy; emerging human data suggest improved HDL function as a surrogate vascular-related endpoint	A relevant bridge between immune control and lipoprotein function, but plaque or ASCVD event reduction remains unproven
Deep immune reset	CD19 CAR-T therapy	Profound B-cell depletion and immune repertoire resetting in refractory SLE	Early studies suggest durable remission in refractory SLE, but no vascular imaging or ASCVD outcome data are available	Transformative for refractory SLE, but cardiovascular protection remains speculative and limited by cost, toxicity, and generalizability
Conventional lipid lowering	Statins	LDL-C reduction with pleiotropic anti-inflammatory effects	Tested in adult and pediatric SLE cohorts; LAPS and APPLE failed to meet primary subclinical atherosclerosis endpoints despite lipid improvement	Remain indicated according to general ASCVD prevention rules, but are unlikely to fully address SLE-specific immune-driven residual risk
Intensive lipid lowering	PCSK9 inhibitors	Potent LDL-C reduction with potential macrophage lipid-inflammation modulation	Strong MACE reduction evidence in general ASCVD populations, but no dedicated SLE-AS trial evidence	Reasonable for very-high-risk SLE patients following general ASCVD frameworks; SLE-specific incremental benefit remains unproven
Plaque inflammatory microenvironment modulation	Canakinumab/IL-1β blockade, colchicine	Targets IL-1β/NLRP3 inflammasome activity, neutrophil-driven inflammation, and sterile plaque inflammation	Event reduction demonstrated in general ASCVD or coronary disease populations, but SLE-AS-specific evidence is lacking	Mechanistically attractive but not ready for routine SLE-AS prevention, especially given infection risk in immunosuppressed SLE patients
Cardiometabolic and endothelial protection	SGLT2 inhibitors, GLP-1 receptor agonists	May improve systemic metabolism, endothelial function, oxidative stress, macrophage inflammation, and cardiorenal risk	Emerging observational or retrospective SLE data mainly involve patients with type 2 diabetes, lupus nephritis, obesity, or metabolic comorbidities; no dedicated SLE plaque endpoint data	Promising for SLE patients with metabolic or cardiorenal risk, but broader SLE-AS vascular benefit remains uncertain

HCQ and CD19 CAR-T therapy are summarized as special categories and are not necessarily plotted in **Figure 2**. AS, atherosclerosis; ASCVD, atherosclerotic cardiovascular disease; BAFF, B-cell activating factor; CAR-T, chimeric antigen receptor T cell; GLP-1 RAs, glucagon-like peptide-1 receptor agonists; HCQ, hydroxychloroquine; IFN, interferon; IL-1β, interleukin-1 beta; JAK–STAT, Janus kinase–signal transducer and activator of transcription; MACE, major adverse cardiovascular events; mTOR, mechanistic target of rapamycin; NAC, N-acetylcysteine; NETs, neutrophil extracellular traps; PCSK9, proprotein convertase subtilisin/kexin type 9; SGLT2, sodium-glucose cotransporter 2; SLE, systemic lupus erythematosus.

## 6 Discussion

The cardiovascular perspective on SLE-associated atherosclerosis is increasingly shifting. Rather than representing the simple coexistence of systemic autoimmunity and a conventional lipid-driven vascular disease, SLE-associated atherosclerosis is better understood as a form of immune–metabolic–vascular remodeling in which persistent interferon signaling, NET-associated oxidative injury, maladaptive myeloid lipid handling, and adaptive immune persistence collectively reshape the arterial wall toward plaque vulnerability and thrombosis. Across these processes, metabolic rewiring appears to function not merely as a bystander, but as a common amplifier linking systemic lupus activity to lesion-level progression. This framework has translational implications. It helps explain why lipid lowering alone or broad immunosuppression alone is often insufficient to fully offset vascular risk in SLE, and why future therapeutic strategies will likely require layered integration of upstream immunometabolic resetting, midstream immune-specific blockade, and downstream vascular-wall protection. At present, however, the major translational gap remains the lack of therapies that are simultaneously well aligned with the SLE-AS disease axis and validated by dedicated vascular imaging or ASCVD outcome studies in SLE. Future work should therefore prioritize biomarker-defined endotypes, vascular surrogate endpoints, and ultimately hard cardiovascular outcomes, so that interventions can be evaluated not only by their ability to control lupus activity, but also by their capacity to modify SLE-associated vascular progression.

## Glossary

2DG: 2-deoxy-D-glucose
ABCA1: ATP-binding cassette transporter A1
ABCG1: ATP binding cassette subfamily G member 1
AMPK: AMP-activated protein kinase
aPL: Antiphospholipid antibodies
ApoA-I: Apolipoprotein A-I
ApoB: Apolipoprotein B
APPLE: Atherosclerosis Prevention in Pediatric Lupus Erythematosus
APS: Antiphospholipid syndrome
AS: Atherosclerosis
ASCVD: Atherosclerotic cardiovascular disease
BAFF: B-cell activating factor
CAC: Coronary artery calcification
CAR-T: Chimeric antigen receptor T cell
cGAS: Cyclic GMP-AMP synthase
CHIP: Clonal hematopoiesis of indeterminate potential
CIMT: Carotid intima-media thickness
CMV: Cytomegalovirus
CRS: Cytokine release syndrome
CXCL: C-X-C motif chemokine ligand
DAMPs: Damage-associated molecular patterns
DCs: Dendritic cells
DN: Double-negative
EBV: Epstein-Barr virus
dsDNA: Double-stranded DNA
eNOS: Endothelial nitric oxide synthase
EPCs: Endothelial progenitor cells
FAO: Fatty acid oxidation
FDA: Food and Drug Administration
GLP-1 RAs: Glucagon-like peptide-1 receptor agonists
HCQ: Hydroxychloroquine
HDL/HDL-C: High-density lipoprotein/High-density lipoprotein cholesterol
HIF-1α: Hypoxia-inducible factor 1 alpha
ICAM-1: Intercellular adhesion molecule 1
ICANS: Immune effector cell-associated neurotoxicity syndrome
IFN-I: Type I interferon
IL-10: Interleukin-10
IL-18: Interleukin-18
IL-1β: Interleukin-1 beta
IMT: Intima-media thickness
ISGs: Interferon-stimulated genes
JAK-STAT: Janus kinase-signal transducer and activator of transcription
LAPS: Lupus Atherosclerosis Prevention Study
LDL/LDL-C: Low-density lipoprotein/Low-density lipoprotein cholesterol
LXR: Liver X receptor
MACE: Major adverse cardiovascular events
MHP: Mitochondrial hyperpolarization
MPO: Myeloperoxidase
mtDNA: Mitochondrial DNA
mTOR: Mechanistic target of rapamycin
mtROS: Mitochondrial reactive oxygen species
NAC: N-acetylcysteine
NETs: Neutrophil extracellular traps
NLRP3: NLR family pyrin domain containing 3
NO: Nitric oxide
oxLDL: Oxidized low-density lipoprotein
OXPHOS: Oxidative phosphorylation
PAMPs: Pathogen-associated molecular patterns
PBMCs: Peripheral blood mononuclear cells
PCSK9: Proprotein convertase subtilisin/kexin type 9
pDCs: Plasmacytoid dendritic cells
PDGFRβ: Platelet-derived growth factor receptor beta
PI3K: Phosphoinositide 3-kinase
piHDL: Pro-inflammatory high-density lipoprotein
PON1: Paraoxonase-1
PRRs: Pattern recognition receptors
RCT: Reverse cholesterol transport
ROS: Reactive oxygen species
SASP: Senescence-associated secretory phenotype
scRNA-seq: Single-cell RNA sequencing
SGLT2i: Sodium-glucose cotransporter 2 inhibitors
SLE: Systemic lupus erythematosus
SMCs: Smooth muscle cells
STING: Stimulator of interferon genes
T2D: Type 2 diabetes
TCR: T cell receptor
TGF-β: Transforming growth factor-beta
Th1/Th17: T helper 1/T helper 17
TLR: Toll-like receptor
TNF-α: Tumor necrosis factor-alpha
Treg: Regulatory T cell
Trm: Tissue-resident memory
USP18: Ubiquitin specific peptidase 18
VCAM-1: Vascular cell adhesion molecule 1
β2-GPI: β2-glycoprotein I.
