# Correction: Systemic lupus erythematosus–driven accelerated atherosclerosis: the immune–metabolic–vascular axis and therapeutic implications

**DOI:** 10.3389/fimmu.2026.1951981

**Published:** 2026-08-06

**Authors:** Meiwei Jiang, FengQi Zhang, MinZhe Ren, ZhiYu Li, ZhiJun Xie, Jing Sun

**Affiliations:** 1 The Second School of Clinical Medicine, Zhejiang Chinese Medical University, Hangzhou, China; 2 Innovative Institute of Chinese Medicine and Pharmacy, Shandong University of Traditional Chinese Medicine, Jinan, China; 3 The First Affiliated Hospital of Zhejiang Chinese Medical University (Zhejiang Provincial Hospital of Chinese Medicine), Hangzhou, China; 4 College of Basic Medical Science, Zhejiang Chinese Medical University, Hangzhou, China; 5 Jinhua Research Institute, Zhejiang Chinese Medical University, Jinhua, China; 6 The Affiliated Lishui Hospital of Traditional Chinese Medicine, Zhejiang Chinese Medical University, Lishui, China

**Keywords:** atherosclerosis, dysfunctional HDL, immunometabolism, neutrophil extracellular traps, systemic lupus erythematosus, type I interferon

Affiliation “Jinhua Research Institute, Zhejiang Chinese Medical University, Jinhua, China” was omitted from the paper. This affiliation has now been added for author(s) ZhiJun Xie.

The original version of this article has been updated.

